# Activation studies with amines and amino acids of the β-carbonic anhydrase encoded by the *Rv3273* gene from the pathogenic bacterium *Mycobacterium tuberculosis*

**DOI:** 10.1080/14756366.2017.1422250

**Published:** 2018-01-11

**Authors:** Andrea Angeli, Sonia Del Prete, Sameh M. Osman, Fatmah A. S. Alasmary, Zeid AlOthman, William A. Donald, Clemente Capasso, Claudiu T. Supuran

**Affiliations:** a Dipartimento Neurofarba, Sezione di Scienze Farmaceutiche e Nutraceutiche, Università degli Studi di Firenze, Florence, Italy;; b CNR, Istituto di Bioscienze e Biorisorse, Napoli, Italy;; c Department of Chemistry, College of Science, King Saud University, Riyadh, Saudi Arabia;; d School of Chemistry, University of New South Wales, Sydney, NSW, Australia

**Keywords:** Carbonic anhydrase, metalloenzymes, pathogens, activators, *Mycobacterium tuberculosis*

## Abstract

The activation of a β-class carbonic anhydrase (CAs, EC 4.2.1.1) from *Mycobacterium tuberculosis*, encoded by the gene Rv3273 (mtCA 3), was investigated using a panel of natural and non-natural amino acids and amines. mtCA 3 was effectively activated by D-DOPA, L-Trp, dopamine and serotonin, with K_A_s ranging between 8.98 and 12.1 µM. L-His and D-Tyr showed medium potency activating effects, with K_A_s in the range of 17.6–18.2 µM, whereas other amines and amino acids were relatively ineffective activators, with K_A_s in the range of 28.9–52.2 µM. As the physiological roles of the three mtCAs present in this pathogen are currently poorly understood and considering that inhibition of these enzymes has strong antibacterial effects, discovering molecules that modulate their enzymatic activity may lead to a better understanding of the factors related to the invasion and colonisation of the host during *Mycobacterium tuberculosis* infection.

## 1. Introduction

Among the bacterial infections which create huge medical problems worldwide, the *Mycobacterium tuberculosis* one is among the most threatening due to a number of causes: (i) it is estimated that one in each three people is latently infected with this pathogen, and although clinical manifestations emerge only in ill, old-aged or immunosuppressed patients, the ease of transmission of this infection creates serious medical challenges^1^^,^^2^; (ii) a large number of *M. tuberculosis* strains became drug resistant or extensively drug resistant to most of almost all clinically used antimycobacterials^1^^,^^2^; (iii) no new such drugs were launched for the last 30 years, which coupled to the general antibiotic resistance of many other pathogenic bacteria, of which *M. tuberculosis* is the tip of the iceberg, may lead to the resurgence of fatal bacterial infections worldwide.

In fact, since the 1950s, it has been considered that the fight against infective diseases caused by bacteria has been a success^1^^,^^2^. However, this does not seem to be the case any longer and new approaches for detecting novel antibacterial drug targets are immediately necessary in order to address this serious medical problem. Proteomics ultimately afforded the possibility to evidence new such drug targets, and among the many proposed proteins are also the bacterial carbonic anhydrases (CAs, EC 4.2.1.1), a family of metalloenzymes involved in crucial steps of the pathogen life cycle^3–6^. In *M. tuberculosis*, three CAs belonging to the β-CA class have been discovered, encoded by the genes Rv1284 (mtCA 1), Rv3588c (mtCA 2) and Rv3273 (mtCA 3)^3–5^. Their inhibition with a variety of compounds such as sulphonamides, phenols and dithiocarbamates (all acting as efficient CA inhibitors)^6–10^ was shown to lead to an impaired growth of the pathogen^5^, allowing us to propose mtCAs as anti-mycobacterial drug targets. Indeed, bacteria encode for CAs belonging to three different genetic families, the α-, β- and γ-CAs^6–8^, and their inhibition was recently investigated in some details in the search of antibiotics with a novel mechanism of action^6^^,^^10–15^. In fact, CA inhibitors (CAIs) of the sulphonamide or sulphamate type, targeting mammalian (human, h) CAs, are in clinical use as diuretics, antiglaucoma, antiepileptic or antiobesity agents for decades^16–18^, whereas more recently their use for the management of hypoxic tumours, neuropathic pain, cerebral ischemia and arthritis started to emerge^19–21^. These diverse applications are due to the fact that at least 15 different α-CA isoforms are present in humans, being involved in critical physiological and pathological processes^16–21^.

In contrast to the CAIs, the CA activators (CAAs) were much less investigated^22^^,^^23^. This type of compounds participates in the CA catalytic cycle, which is shown schematically in the following equations:

(1) $$\begin{array}{c} {\text{H}}_{\text{2}}\text{O}\\ {\text{EZn}}^{\text{2}+}-{\text{OH}}^{-}+{\text{ CO}}_{\text{2}}\Leftrightarrow {\text{EZn}}^{\text{2}+}-{\text{HCO}}_{\text{3}}^{-}\Leftrightarrow {\text{EZn}}^{\text{2}+}-{\text{OH}}_{\text{2}}+{\text{HCO}}_{\text{3}}^{-} \end{array}$$

(2) $${\text{EZn}}^{\text{2}+}-{\text{OH}}_{\text{2}}\Leftrightarrow {\text{EZn}}^{\text{2}+}-{\text{HO}}^{-}+{\text{ H}}^{+}$$

The first step involves the nucleophilic attack of a zinc-bound hydroxide species of the enzyme on the CO_2_ substrate, bound in a hydrophobic pocket nearby and optimally orientated for the hydration reaction (Equation (1))^7^^,^^8^. Bicarbonate formed in the hydration reaction is then replaced by an incoming water molecule, with the generation of the catalytically acid form of the enzyme, EZn^2+^−OH_2_ (Equation (1)). For the regeneration of the zinc hydroxide species, a proton transfer reaction occurs from the Zn(II)-bound water molecule to the external medium (Equation (2)), which is the rate-determining step of the entire catalytic cycle:

(3) $$\begin{array}{c} {\text{EZn}}^{\text{2}+}-{\text{OH}}_{\text{2}}+\text{A}\Leftrightarrow [{\text{EZn}}^{\text{2}+}-{\text{OH}}_{\text{2}}-\text{A}]\Leftrightarrow [{\text{EZn}}^{\text{2}+}-{\text{HO}}^{-}-{\text{ AH}}^{+}]\Leftrightarrow {\text{EZn}}^{\text{2}+}-{\text{HO}}^{-}+{\text{AH}}^{+}\\ \text{enzyme }-\text{ activator complexes} \end{array}$$

In the presence of activators (A in Equation (3)), the formation of enzyme–activator complexes occurs, in which the proton transfer reaction became intramolecular, being thus more efficient than the corresponding intermolecular process^7^^,^^8^. This mechanism of CA activation was demonstrated by kinetic and crystallographic studies for the human isoforms hCA I and II^9^. The activator-binding site was shown to be situated at the entrance of the active site cavity. Most of the activators belong to the amino and/or amino acid chemotypes, and possess moieties with an appropriate p*K*a (generally in the range of 6–8) for efficient proton shuttling processes between the active site and the environment^7–9^.

CAAs were extensively investigated in the last period for their interaction with all human CAs^24–30^, but their effects on bacterial enzymes were poorly studied up until now^31–33^. The same situation is in fact valid for the investigation of bacterial CA inhibitors, ad mentioned above^31–33^.

Considering the fact that few CA activation studies of bacterial enzymes are available, and none of them for the mycobacterial enzymes, here we report the first such study in which we evaluated the activation of mtCA 3 (encoded by the gene Rv3273) with a panel of amines and amino acid derivatives.

## 2. Materials and methods

### 2.1. Materials

Amino acids and amines **1–19** were commercially available, highest purity reagents from Sigma-Aldrich, Milan, Italy. Rv3273 was a recombinant protein produced as reported earlier by our group^4^^,^^5^.

### 2.2. CA enzyme activation assay

An Sx.18Mv-R Applied Photophysics (Oxford, UK) stopped-flow instrument has been used to assay the catalytic activity of various CA isozymes for CO_2_ hydration reaction^34^. Phenol red (at a concentration of 0.2 mM) was used as indicator, working at the absorbance maximum of 557 nm, with 10 mM Hepes (pH 7.5) or TRIS (pH 8.3) as buffers, 0.1 M Na_2_SO_4_ (for maintaining constant ionic strength), following the CA-catalysed CO_2_ hydration reaction for a period of 10 s at 25 °C. Activity of the α-CAs was measured at pH 7.5 whereas that of the β-class enzymes at pH 8.3 in order to avoid the possibility that their active site is closed^3^. The CO_2_ concentrations ranged from 1.7 to 17 mM for the determination of the kinetic parameters and activation constants. For each activator at least six traces of the initial 5–10% of the reaction have been used for determining the initial velocity. The uncatalysed rates were determined in the same manner and subtracted from the total observed rates. Stock solutions of activators (10 mM) were prepared in distilled–deionised water and dilutions up to 1 nM were done thereafter with the assay buffer. Activator and enzyme solutions were pre-incubated together for 15 min (standard assay at room temperature) prior to assay, in order to allow for the formation of the E–A complex. The activation constant (K_A_), defined similarly with the inhibition constant K_I_, can be obtained by considering the classical Michaelis–Menten equation (Equation (4)), which has been fitted by non-linear least squares by using PRISM 3:

(4) $$\text{v }={\text{v}}_{\text{max}}/\left\{\text{1}+{\text{K}}_{\text{M}}/\left[\text{S}\right]\left(\text{1}+{\left[\text{A}\right]}_{\text{f}}/{\text{K}}_{\text{A}}\right)\right\}$$

where [A]_f_ is the free concentration of activator.

Working at substrate concentrations considerably lower than K_M_ ([S] **≪**K_M_), and considering that [A]_f_ can be represented in the form of the total concentration of the enzyme ([E]_t_) and activator ([A]_t_), the obtained competitive steady-state equation for determining the activation constant is given by the following equation:

(5) $$\text{v}={\text{v}}_{0}.{\text{K}}_{\text{A}}/\left\{{\text{K}}_{\text{A}}+{\left({\left[\text{A}\right]}_{\text{t}}-0.\text{5}\{\left({\left[\text{A}\right]}_{\text{t}}+{\left[\text{E}\right]}_{\text{t}}+{\text{K}}_{\text{A}}\right)-{\left({\left[\text{A}\right]}_{\text{t}}+{\left[\text{E}\right]}_{\text{t}}+{\text{K}}_{\text{A}}\right)}^{\text{2}}-\text{4}{\left[\text{A}\right]}_{\text{t}}.{\left[\text{E}\right]}_{\text{t}}\right)}^{\text{1}/\text{2}}\right\}\}$$

where v_0_ represents the initial velocity of the enzyme-catalysed reaction in the absence of activator^23–30^.

## 3. Results and discussion

**Figure 1.**
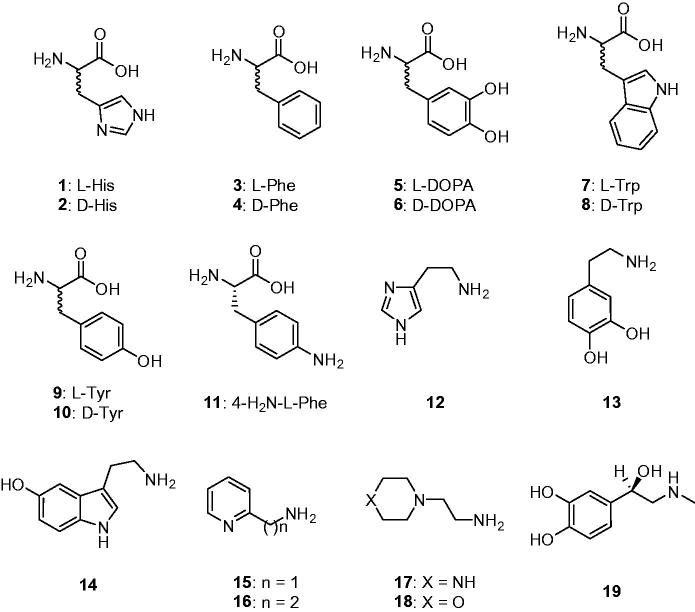
Amino acids **1–11** and amines **12–19** investigated as mtCA 3 activators.

Natural and non-natural amino acids and amines **1–19** were included among the investigated compounds (Figure 1). These activators were included in this study, as they were employed for investigations as CAAs against many classes of CAs, including the few bacterial ones investigated so far^33^.

Data of Table 1 show that l-Trp (at 10 µ.M concentration), which is a medium potency activator for all enzymes considered here, i.e. hCA I, II and mtCA 3, enhanced k_cat_ values for all of them, whereas K_M_ remained unchanged, a situation observed for all CAAs investigated so far, both for those belonging to vertebrates (α-class enzymes) and micro-organism (enzymes belonging to various CA genetic families)^23–30^^,^^33^. l-Trp was a micromolar activator for all these enzymes with K_A_s in the range of 27–44 µM for hCA I and II, and with a K_A_ of 8.98 µM against mtCA 3 (Table 1). l-Trp induced an increase of the kinetic constant of hCA I and II compared with the uncatalysed rate (of 1.7–3.5 times, which for such efficient enzymes is highly significant). For the mtCA 3, a similar kinetic effect was observed, with an increase of k_cat_ of 3.2 times in the presence of 10 µM l-Trp (compared with the rate in the absence of activator). We stress again, K_M_ remained the same in the presence and absence of activator, which proves that the substrate and activator binding sites are different (this has been confirmed by X-ray crystallography for several α-CAs complexed with activators)^23–30^.

**Table 1.** Activation of human carbonic anhydrase (hCA) isozymes I, II and mtCA 3 with l-Trp, at 25 °C, for the CO_2_ hydration reaction^34^.

Isozyme	k_cat_^c^ (s^−1^)	K_M_^c^ (mM)	(k_cat_)_l-Trp_^d^ (s^−1^)	K_A_^e^ (μM) l-Trp
hCA I^a^	2.0 × 10^5^	4.0	3.4 × 10^5^	44
hCA II^a^	1.4 × 10^6^	9.3	4.9 × 10^6^	27
mtCA 3^b^	4.3 × 10^5^	10.7	13.8 × 10^5^	8.98

a Human recombinant isozymes, from Ref.^23^.

b Bacterial recombinant enzyme, this work.

c Observed catalytic rate without activator. K_M_ values in the presence and the absence of activators were the same for the various CAs (data not shown).

b Observed catalytic rate in the presence of 10 μM activator.

c The activation constant (K_A_) for each enzyme was obtained by fitting the observed catalytic enhancements as a function of the activator concentration.^11^ Mean from at least three determinations by a stopped-flow, CO_2_ hydrase method^34^. Standard errors were in the range of 5–10% of the reported values (data not shown).

Amino acids and amines **1–19** (Figure 1) previously investigated as CAAs of human (α-class) CAs^23^ and for the activation of few bacterial enzymes^33^ showed significant activating effects against mtCA 3, as observed from data of Table 2, in which the activation constants (K_A_s) of these compounds against three CAs are presented (hCA I and II data are included for comparison reasons)^23^. The following structure-activity relationship (SAR) can be evidenced from the data of Table 2:

**Table 2.** Activation constants of hCA I, hCA II and the bacterial mtCA 3 with amino acids and amines **1–19**. Data for hCA I and II are from Ref.^23^.

		K_A_ (μM)^c^		
No.	Compound	hCA I^a^	hCA II^a^	mtCA 3^b^
**1**	l-His	0.03	10.9	18.2
**2**	d-His	0.09	43	32.5
**3**	l-Phe	0.07	0.013	30.6
**4**	d-Phe	86	0.035	44.1
**5**	l-DOPA	3.1	11.4	30.0
**6**	d-DOPA	4.9	7.8	9.74
**7**	l-Trp	44	27	8.98
**8**	d-Trp	41	12	43.7
**9**	l-Tyr	0.02	0.011	28.9
**10**	d-Tyr	0.04	0.013	17.6
**11**	4-H_2_N-L-Phe	0.24	0.15	40.5
**12**	Histamine	2.1	125	34.2
**13**	Dopamine	13.5	9.2	12.1
**14**	Serotonin	45	50	10.3
**15**	2-Pyridyl-methylamine	26	34	43.3
**16**	2-(2-Aminoethyl)pyridine	13	15	45.9
**17**	1-(2-Aminoethyl)-piperazine	7.4	2.3	50.3
**18**	4-(2-Aminoethyl)-morpholine	0.14	0.19	52.0
**19**	l-Adrenaline	0.09	96	52.2

a Human recombinant isozymes, stopped flow CO_2_ hydrase assay method^23^.

b This work.

c Mean from three determinations by a stopped-flow, CO_2_ hydrase method^34^. Standard errors were in the range of 5–10% of the reported values (data not shown).

- The most effective mtCA 3 activators were d-DOPA, l-Trp, dopamine and serotonin, with K_A_s ranging between 8.98 and 12.1 µM. Thus, both amino acid and amine types of activators show efficient activating effects on mtCA 3.
- l-His and d-Tyr showed medium potency activating effects, with K_A_s in the range of 17.6–18.2 µM.
- The remaining derivatives showed a weaker mtCA 3 activation potency, with K_A_s in the range of 28.9–52.2 µM. The SAR is thus rather well defined. For example, with few exceptions the l-amino acids were more effective mtCA 3 activators compared with the corresponding d-enantiomer. The exceptions are d-DOPA and d-Tyr which were more effective mtCA 3 activators compared with the corresponding l-enantiomer. Amines (with the exception of dopamine and serotonin) were generally less effective mtCA 3 activators compared with structurally related amino acid derivatives (compare histamine and l-/d-His; l-adrenaline and l-/d-DOPA, etc.), but the differences were not very important. In fact, no submicromolar mtCA 3 activators were detected in this study.
- There were important differences of activity for these CAAs against the human isoforms hCA I and II compared to the mycobacterial enzyme mtCA 3. Only l-Trp and serotonin were better activators of the bacterial versus the human isoforms, whereas all other compounds were more effective (sometimes in the nanomolar range) for activating the human CAs (Table 2).

## 4. Conclusions

The first activation study of a mycobacterial CA is reported here. mtCA 3 was effectively activated by D-DOPA, l-Trp, dopamine and serotonin, with K_A_s ranging between 8.98 and 12.1 µM. l-His and d-Tyr showed medium potency activating effects, with K_A_s in the range of 17.6–18.2 µM, whereas other amines and amino acids were weakly effective activators, with K_A_s in the range of 28.9–52.2 µM. As the physiological role of the three mtCAs is poorly understood at this moment and the inhibition of such enzymes was shown to lead to strong antibacterial effects, modulating the activity of these CAs may lead to a better understanding of factors connected to the invasion and colonisation of the host during *Mycobacterium tuberculosis* infection.

## References

[1] a) Singh V, Dhar N, Pató J, et al Identification of aminopyrimidine-sulfonamides as potent modulators of Wag31-mediated cell elongation in mycobacteria. Mol Microbiol 2017;103:13–25. b) Jones PB, Parrish NM, Houston TA, et al A new class of antituberculosis agents. J Med Chem 2000;43:3304–14. 10.1111/mmi.13535 27677649

[2] a) Borrell S, Trauner A. Strain diversity and the evolution of antibiotic resistance. Adv Exp Med Biol 2017;1019:263–79. b) Al-Humadi HW, Al-Saigh RJ, Al-Humadi AW, Addressing the challenges of tuberculosis: a brief historical account. Front Pharmacol 2017;8:689 c) Cadena AM, Fortune SM, Flynn JL. Heterogeneity in tuberculosis. Nat Rev Immunol 2017;17:691–702.

[3] a) Suarez Covarrubias A, Larsson AM, Högbom M, et al Structure and function of carbonic anhydrases from *Mycobacterium tuberculosis*. J Biol Chem 2005;280:18782–9. b) Covarrubias AS, Bergfors T, Jones TA, Högbom M. Structural mechanics of the pH-dependent activity of beta-carbonic anhydrase from *Mycobacterium tuberculosis*. J Biol Chem 2006;281:4993–9. 10.1074/jbc.M414348200 15753099

[4] a) Nishimori I, Minakuchi T, Maresca A, et al The β-carbonic anhydrases from *Mycobacterium tuberculosis* as drug targets. Curr Pharm Des 2010;16:3300–9. b) Nishimori I, Minakuchi T, Vullo D, et al Carbonic anhydrase inhibitors. Cloning, characterization, and inhibition studies of a new beta-carbonic anhydrase from *Mycobacterium tuberculosis*. J Med Chem 2009;52:3116–20. c) Güzel O, Maresca A, Scozzafava A, et al Discovery of low nanomolar and subnanomolar inhibitors of the mycobacterial beta-carbonic anhydrases Rv1284 and Rv3273. J Med Chem 2009;52:4063–7.

[5] a) Maresca A, Carta F, Vullo D, et al Carbonic anhydrase inhibitors. Inhibition of the Rv1284 and Rv3273 beta-carbonic anhydrases from *Mycobacterium tuberculosis* with diazenylbenzenesulfonamides. Bioorg Med Chem Lett 2009;19:4929–32. b) Carta F, Maresca A, Covarrubias AS, et al Carbonic anhydrase inhibitors. Characterization and inhibition studies of the most active beta-carbonic anhydrase from *Mycobacterium tuberculosis*, Rv3588c. Bioorg Med Chem Lett 2009;19:6649–54. c) Aspatwar A, Hammarén M, Koskinen S, et al β-CA-specific inhibitor dithiocarbamate Fc14-584B: a novel antimycobacterial agent with potential to treat drug-resistant tuberculosis. J Enzyme Inhib Med Chem 2017;32:832–40.

[6] a) Capasso C, Supuran CT. An overview of the alpha-, beta-and gamma-carbonic anhydrases from Bacteria: can bacterial carbonic anhydrases shed new light on evolution of bacteria? J Enzyme Inhib Med Chem 2015;30:325–32. b) Del Prete S, Vullo D, Fisher GM, et al Discovery of a new family of carbonic anhydrases in the malaria pathogen *Plasmodium falciparum* – the η-carbonic anhydrases. Bioorg Med Chem Lett 2014;24:4389–96. c) Supuran CT. Structure-based drug discovery of carbonic anhydrase inhibitors. J Enzyme Inhib Med Chem 2012;27:759–72.

[7] a) Supuran CT. Carbonic anhydrases: from biomedical applications of the inhibitors and activators to biotechnological use for CO_2_ capture. J Enzyme Inhib Med Chem 2013;28:229–30. b) Supuran CT. How many carbonic anhydrase inhibition mechanisms exist? J Enzyme Inhib Med Chem 2016;31:345–60. c) Alterio V, Di Fiore A, D’Ambrosio K, et al Multiple binding modes of inhibitors to carbonic anhydrases: how to design specific drugs targeting 15 different isoforms? Chem Rev 2012;112:4421–68. d) Abbate F, Winum JY, Potter BV, et al Carbonic anhydrase inhibitors: X-ray crystallographic structure of the adduct of human isozyme II with EMATE, a dual inhibitor of carbonic anhydrases and steroid sulfatase. Bioorg Med Chem Lett 2004;14:231–4.

[8] a) Supuran CT. Advances in structure-based drug discovery of carbonic anhydrase inhibitors. Expert Opin Drug Discov 2017;12:61–88. b) Supuran CT. Structure and function of carbonic anhydrases. Biochem J 2016;473:2023–32. c) Supuran CT. Carbonic anhydrases: novel therapeutic applications for inhibitors and activators. Nat Rev Drug Discov 2008;7:168–81. d) Neri D, Supuran CT. Interfering with pH regulation in tumours as a therapeutic strategy. Nat. Rev. Drug Discov 2011;10:767–77. e) Supuran CT, Vullo D, Manole G, et al Designing of novel carbonic anhydrase inhibitors and activators. Curr Med Chem Cardiovasc Hematol Agents 2004;2:49–68.

[9] a) Briganti F, Mangani S, Orioli P, et al Carbonic anhydrase activators: X-ray crystallographic and spectroscopic investigations for the interaction of isozymes I and II with histamine. Biochemistry 1997;36:10384–92. b) Clare BW, Supuran CT. Carbonic anhydrase activators. 3: structure–activity correlations for a series of isozyme II activators. J Pharm Sci 1994;83:768–73. c) Ilies M, Scozzafava A, Supuran CT. Carbonic anhydrase activators In: Supuran CT, Scozzafava A, Conway J eds. Carbonic anhydrase – its inhibitors and activators. Boca Raton (FL): CRC Press; 2004: 317–52. 10.1021/bi970760v 9265618

[10] a) Scozzafava A, Menabuoni L, Mincione F, Supuran CT. Carbonic anhydrase inhibitors. A general approach for the preparation of water-soluble sulfonamides incorporating polyamino − polycarboxylate tails and of their metal complexes possessing long-lasting, topical intraocular pressure-lowering properties. J Med Chem 2002;45:1466–76. b) Pacchiano F, Aggarwal M, Avvaru BS, et al Selective hydrophobic pocket binding observed within the carbonic anhydrase II active site accommodate different 4-substituted-ureido-benzenesulfonamides and correlate to inhibitor potency. Chem Commun (Camb) 2010;46:8371–3. c) Carta F, Scozzafava A, Supuran CT. Sulfonamides: a patent review (2008–2012). Expert Opin Ther Pat 2012;22:747–58. d) Puccetti L, Fasolis G, Vullo D, et al Carbonic anhydrase inhibitors. Inhibition of cytosolic/tumor-associated carbonic anhydrase isozymes I, II, IX, and XII with Schiff's bases incorporating chromone and aromatic sulfonamide moieties, and their zinc complexes. Bioorg Med Chem Lett 2005;15:3096–101.

[11] a) Akocak S, Lolak N, Vullo D, et al Synthesis and biological evaluation of histamine Schiff bases as carbonic anhydrase I, II, IV, VII, and IX activators. J Enzyme Inhib Med Chem 2017;32:1305–12. b) Angeli A, Vaiano F, Mari F, et al Psychoactive substances belonging to the amphetamine class potently activate brain carbonic anhydrase isoforms VA, VB, VII, and XII. J Enzyme Inhib Med Chem 2017; 3:1253–9. c) Licsandru E, Tanc M, Kocsis I, et al A class of carbonic anhydrase I – selective activators. J Enzyme Inhib Med Chem 2017;32:37–46.

[12] a) Kumar R, Sharma V, Bua S, et al Synthesis and biological evaluation of benzenesulphonamide-bearing 1,4,5-trisubstituted-1,2,3-triazoles possessing human carbonic anhydrase I, II, IV, and IX inhibitory activity. J Enzyme Inhib Med Chem 2017;32:1187–94. b) Del Prete S, Perfetto R, Rossi M, et al A one-step procedure for immobilising the thermostable carbonic anhydrase (SspCA) on the surface membrane of *Escherichia coli*. J Enzyme Inhib Med Chem 2017; 3:1120–8. c) Stanica L, Gheorghiu M, Stan M, et al Quantitative assessment of specific carbonic anhydrase inhibitors effect on hypoxic cells using electrical impedance assays. J Enzyme Inhib Med Chem 2017;32:1079–90.

[13] a) Zengin Kurt B, Sonmez F, Durdagi S, et al Synthesis, biological activity and multiscale molecular modeling studies for coumaryl-carboxamide derivatives as selective carbonic anhydrase IX inhibitors. J Enzyme Inhib Med Chem 2017;32:1042–52. b) Nocentini A, Vullo D, Del Prete S, et al Inhibition of the β-carbonic anhydrase from the dandruff-producing fungus Malassezia globosa with monothiocarbamates. J Enzyme Inhib Med Chem 2017;3:1064–70. c) Del Prete S, Vullo D, Osman SM, et al Anion inhibitors of the β-carbonic anhydrase from the pathogenic bacterium responsible of tularemia, *Francisella tularensis*. Bioorg Med Chem 2017;25:4800–4.

[14] a) Perfetto R, Del Prete S, Vullo D, et al Cloning, expression and purification of the α-carbonic anhydrase from the mantle of the Mediterranean mussel, *Mytilus galloprovincialis*. J Enzyme Inhib Med Chem 2017;32:1029–35. b) Abdoli M, Angeli A, Bozdag M, et al Synthesis and carbonic anhydrase I, II, VII, and IX inhibition studies with a series of benzo[d]thiazole-5- and 6-sulfonamides. J Enzyme Inhib Med Chem 2017;32:1071–8. c) De Simone G, Langella E, Esposito D, et al Insights into the binding mode of sulphamates and sulphamides to hCA II: crystallographic studies and binding free energy calculations. J Enzyme Inhib Med Chem 2017;32:1002–11. d) Scozzafava A, Menabuoni L, Mincione F, et al Carbonic anhydrase inhibitors: perfluoroalkyl/aryl-substituted derivatives of aromatic/heterocyclic sulfonamides as topical intraocular pressure-lowering agents with prolonged duration of action. J Med Chem 2000;43:4542–51.

[15] a) Supuran CT, Capasso C. Carbonic anhydrase from *Porphyromonas gingivalis* as a drug target. Pathogens 2017;6:E30 b) Capasso C, Supuran CT. inhibition of bacterial carbonic anhydrases as a novel approach to escape drug resistance. Curr Top Med Chem 2017;17:1237–48. c) Supuran CT, Capasso C. New light on bacterial carbonic anhydrases phylogeny based on the analysis of signal peptide sequences. J Enzyme Inhib Med Chem 2016;31:1254–60.

[16] Carta F, Supuran CT. Diuretics with carbonic anhydrase inhibitory action: a patent and literature review (2005–2013). Expert Opin Ther Pat 2013;23:681–91. 23488823 10.1517/13543776.2013.780598

[17] Masini E, Carta F, Scozzafava A, Supuran CT. Antiglaucoma carbonic anhydrase inhibitors: a patent review. Expert Opin Ther Pat 2013;23:705–16. 23627893 10.1517/13543776.2013.794788

[18] Scozzafava A, Supuran CT, Carta F. Antiobesity carbonic anhydrase inhibitors: a literature and patent review. Expert Opin Ther Pat 2013;23:725–35. 23607332 10.1517/13543776.2013.790957

[19] a) Monti SM, Supuran CT, De Simone G. Anticancer carbonic anhydrase inhibitors: a patent review (2008–2013). Expert Opin Ther Pat 2013;23:737–49. b) Supuran CT. Carbonic anhydrase inhibition and the management of hypoxic tumors. Metabolites 2017;7:E48 c) Ward C, Langdon SP, Mullen P, et al New strategies for targeting the hypoxic tumour microenvironment in breast cancer. Cancer Treat Rev 2013;39:171–9. d) Garaj V, Puccetti L, Fasolis G, et al Carbonic anhydrase inhibitors: novel sulfonamides incorporating 1,3,5-triazine moieties as inhibitors of the cytosolic and tumour-associated carbonic anhydrase isozymes I, II and IX. Bioorg Med Chem Lett 2005;15:3102–8.

[20] a) Supuran CT. Carbonic anhydrase inhibition and the management of neuropathic pain. Expert Rev Neurother 2016;16:961–8. b) Di Cesare Mannelli L, Micheli L, Carta F, et al Carbonic anhydrase inhibition for the management of cerebral ischemia: in vivo evaluation of sulfonamide and coumarin inhibitors. J Enzyme Inhib Med Chem 2016;31:894–9. 10.3109/14756366.2015.1113407 26607399

[21] a) Margheri F, Ceruso M, Carta F, et al Overexpression of the transmembrane carbonic anhydrase isoforms IX and XII in the inflamed synovium. J Enzyme Inhib Med Chem 2016;31(sup4):60–3. b) Bua S, Di Cesare Mannelli L, Vullo D, et al Design and synthesis of novel nonsteroidal anti-inflammatory drugs and carbonic anhydrase inhibitors hybrids (NSAIDs-CAIs) for the treatment of rheumatoid arthritis. J Med Chem 2017;60:1159–70.

[22] a) Tu C, Rowlett RS, Tripp BC, et al Chemical rescue of proton transfer in catalysis by carbonic anhydrases in the beta- and gamma-class. Biochemistry 2002;41:15429–35. b) Smith KS, Ingram-Smith C, Ferry JG. Roles of the conserved aspartate and arginine in the catalytic mechanism of an archaeal beta-class carbonic anhydrase. J Bacteriol 2002;184:4240–5. 10.1021/bi026831u 12484784

[23] a) Temperini C, Scozzafava A, Supuran CT. Carbonic anhydrase activation and the drug design. Curr Pharm Des 2008;14:708–15. b) Isik S, Guler OO, Kockar F, et al Saccharomyces cerevisiae β-carbonic anhydrase: inhibition and activation studies. Curr Pharm Des 2010;16:3327–36. 10.2174/138161208783877857 18336317

[24] a) Temperini C, Scozzafava A, Vullo D, Supuran CT. Carbonic anhydrase activators. Activation of isozymes I, II, IV, VA, VII, and XIV with l- and d-histidine and crystallographic analysis of their adducts with isoform II: engineering proton-transfer processes within the active site of an enzyme. Chemistry 2006;12:7057–66. b) Temperini C, Scozzafava A, Vullo D, Supuran CT. Carbonic anhydrase activators. Activation of isoforms I, II, IV, VA, VII, and XIV with l- and d-phenylalanine and crystallographic analysis of their adducts with isozyme II: stereospecific recognition within the active site of an enzyme and its consequences for the drug design. J Med Chem 2006;49:3019–27. c) Temperini C, Innocenti A, Scozzafava A, Supuran CT. Carbonic anhydrase activators: kinetic and X-ray crystallographic study for the interaction of d- and l-tryptophan with the mammalian isoforms I-XIV. Bioorg Med Chem 2008;16:8373–8.

[25] a) Temperini C, Innocenti A, Scozzafava A, et al Carbonic anhydrase activators: l-adrenaline plugs the active site entrance of isozyme II, activating better isoforms I, IV, VA, VII, and XIV. Bioorg Med Chem Lett 2007;17:628–35. b) Temperini C, Scozzafava A, Puccetti L, Supuran CT. Carbonic anhydrase activators: X-ray crystal structure of the adduct of human isozyme II with l-histidine as a platform for the design of stronger activators. Bioorg Med Chem Lett 2005;15:5136–41. c) Temperini C, Scozzafava A, Supuran CT. Carbonic anhydrase activators: the first X-ray crystallographic study of an adduct of isoform I. Bioorg Med Chem Lett 2006;16:5152–6.

[26] a) Vullo D, Nishimori I, Innocenti A, et al Carbonic anhydrase activators: an activation study of the human mitochondrial isoforms VA and VB with amino acids and amines. Bioorg Med Chem Lett. 2007; 17:1336–40. b) Pastorekova S, Vullo D, Nishimori I, et al Carbonic anhydrase activators: activation of the human tumor-associated isozymes IX and XII with amino acids and amines. Bioorg Med Chem 2008;16:3530–6. c) Nishimori I, Onishi S, Vullo D, et al Carbonic anhydrase activators. The first activation study of the human secretory isoform VI. Bioorg Med Chem 2007;15:5351–7.

[27] a) Parkkila S, Vullo D, Puccetti L, et al Carbonic anhydrase activators: activation of isozyme XIII with amino acids and amines. Bioorg Med Chem Lett 2006; 1: 3955–9. b) Vullo D, Innocenti A, Nishimori I, et al Carbonic anhydrase activators: activation of the human isoforms VII (cytosolic) and XIV (transmembrane) with amino acids and amines. Bioorg Med Chem Lett 2007;1:4107–12. c) Vullo D, Nishimori I, Scozzafava A, Supuran CT. Carbonic anhydrase activators: activation of the human cytosolic isozyme III and membrane-associated isoform IV with amino acids and amines. Bioorg Med Chem Lett 2008;18:4303–7.

[28] a) Innocenti A, Hilvo M, Parkkila S, et al Carbonic anhydrase activators. Activation of the membrane-associated isoform XV with amino acids and amines. Bioorg Med Chem Lett 2009;19:3430–3. b) Supuran CT, Dinculescu A, Balaban AT. Carbonic anhydrase activators. Part 5. CA II activation by 2,4,6-trisubstituted pyridinium cations with 1-(ω-aminoalkyl) side chains. Rev Roum Chim 1993;38:343–9. c) Supuran CT, Barboiu M, Luca C, et al Carbonic anhydrase activators. Part 14. Synthesis of mono- and bis-pyridinium salt derivatives of 2-amino-5-(2-aminoethyl)- and 2-amino-5-(3-aminopropyl)-1,3,4-thiadiazole, and their interaction with isozyme II. Eur J Med Chem 1996;31:597–606. d) Ilies MA, Banciu MD, Ilies M, et al Carbonic anhydrase activators. Part 17. Synthesis and activation study of a series of 1-(1,2,4-triazole-(1H)-3-yl)-2,4,6-trisubstituted-pyridinium salts against isozymes I, II and IV. Eur J Med Chem 1997;32:911–8.

[29] a) Ilies M, Banciu MD, Ilies MA, et al Carbonic anhydrase activators: design of high affinity isozymes I, II, and IV activators, incorporating tri-/tetrasubstituted-pyridinium-azole moieties. J Med Chem 2002;45:504–10. b) Dave K, Scozzafava A, Vullo D, et al Pyridinium derivatives of histamine are potent activators of cytosolic carbonic anhydrase isoforms I, II and VII. Org Biomol Chem 2011;9:2790–800. c) Dave K, Ilies MA, Scozzafava A, et al An inhibitor-like binding mode of a carbonic anhydrase activator within the active site of isoform II. Bioorg Med Chem Lett 2011;21:2764–8.

[30] a) Scozzafava A, Supuran CT. Carbonic anhydrase activators: human isozyme II is strongly activated by oligopeptides incorporating the carboxyterminal sequence of the bicarbonate anion exchanger AE1. Bioorg Med Chem Lett 2002;12:1177–80. b) Scozzafava A, Supuran CT. Carbonic anhydrase activators: high affinity isozymes I, II, and IV activators, incorporating a beta-alanyl-histidine scaffold. J Med Chem 2002;45:284–91. c) Abdo MR, Vullo D, Saada MC, et al Carbonic anhydrase activators: activation of human isozymes I, II and IX with phenylsulfonylhydrazido l-histidine derivatives. Bioorg Med Chem Lett 2009;19:2440–3. d) Saada MC, Montero JL, Vullo D, et al Carbonic anhydrase activators: gold nanoparticles coated with derivatized histamine, histidine, and carnosine show enhanced activatory effects on several mammalian isoforms. J Med Chem 2011;5:1170–7. e) Zhang Y, Legrand YM, Petit E, et al Dynamic encapsulation and activation of carbonic anhydrase in multivalent dynameric host matrices. Chem Commun (Camb) 2016;52:4053–5.

[31] Vullo D, Kumar RSS, Scozzafava A, et al Sulphonamide inhibition studies of the β-carbonic anhydrase from the bacterial pathogen *Clostridium perfringens*. J Enzyme Inhib Med Chem 2018;33:31–6. 29098923 10.1080/14756366.2017.1388233 PMC6009973

[32] a) Modak JK, Liu YC, Supuran CT, Roujeinikova A. Structure–activity relationship for sulfonamide inhibition of *Helicobacter pylori* α-carbonic anhydrase. J Med Chem 2016;59:11098–109. b) Cau Y, Mori M, Supuran CT, Botta M. Mycobacterial carbonic anhydrase inhibition with phenolic acids and esters: kinetic and computational investigations. Org Biomol Chem 2016;14:8322–30. c) Supuran CT. Bortezomib inhibits bacterial and fungal β-carbonic anhydrases. Bioorg Med Chem 2016;24:4406–9. d) Supuran CT. Legionella pneumophila carbonic anhydrases: underexplored antibacterial drug targets. Pathogens 2016;5:E44 e) Supuran CT, Nicolae A, Popescu A. Carbonic anhydrase inhibitors. Part 35. Synthesis of Schiff bases derived from sulfanilamide and aromatic aldehydes: the first inhibitors with equally high affinity towards cytosolic and membrane-bound isozymes. Eur J Med Chem 1996;31:431–8.

[33] a) Vullo D, De Luca V, Scozzafava A, et al The first activation study of a bacterial carbonic anhydrase (CA). The thermostable α-CA from *Sulfurihydrogenibium yellowstonense* YO3AOP1 is highly activated by amino acids and amines. Bioorg Med Chem Lett 2012;22:6324–7. b) Innocenti A, Zimmerman SA, Scozzafava A, et al Carbonic anhydrase activators: activation of the archaeal beta-class (Cab) and gamma-class (Cam) carbonic anhydrases with amino acids and amines. Bioorg Med Chem Lett 2008;18:6194–8. c) Vullo D, Del Prete S, Osman SM, et al Comparison of the amine/amino acid activation profiles of the β- and γ-carbonic anhydrases from the pathogenic bacterium *Burkholderia pseudomallei*. J Enzyme Inhib Med Chem 2018;33:25–30. d) Vullo D, Del Prete S, Osman SM, et al. *Burkholderia pseudomallei* γ-carbonic anhydrase is strongly activated by amino acids and amines. Bioorg Med Chem Lett 2017;27:77–80.

[34] Khalifah RG. The carbon dioxide hydration activity of carbonic anhydrase. I. Stop-flow kinetic studies on the native human isoenzymes B and C. J Biol Chem 1971;246:2561–73. 4994926

